# Supplementary material for: The Kitty Microbiome Project: Defining the Healthy Fecal “Core Microbiome” in Pet Domestic Cats
Source: Vet Sci. 2022 Nov 16;9(11):635. doi: 10.3390/vetsci9110635 (PMC9698023; doi:10.3390/vetsci9110635)

SUPPLEMENTARY FIGURES FOR:

The Kitty Microbiome Project: Defining the Healthy Fecal "Core Microbiome" in Pet Domestic Cats

Holly H. Ganz[1,*], Guillaume Jospin[1], Connie A. Rojas[2], Alex L. Martin[1], Katherine Dahlhausen[1], Dawn D. Kingsbury[1], Carlton X. Osborne[1], Zhandra Entrolezo[1], Syd Redner[1], Bryan Ramirez[1], Jonathan A. Eisen[2], Madeleine Leahy[2], Chase Keaton[2], Janine Wong[2], Jennifer Gardy[3], Jessica K. Jarett[1]

[1] AnimalBiome, 400 29th Street, Suite 101, Oakland, CA 94609, USA
[2] Department of Evolution and Ecology, University of California, Davis, CA 95616, USA
[3] Bill & Melinda Gates Foundation, Seattle, WA 98109, USA

Name and email address of corresponding author:
Holly H. Ganz (holly@animalbiome.com)

List of Supplemental Figures

Figure S1 Histograms of core genera abundances across samples
Figure S2 Bacterial genera differentially abundant among age classes
Figure S3 Bacterial genera differentially abundant among diet components
Figure S4 Heatmap of taxa used by Random Forest for differentiating among diet types
Figure S5 Heatmap of taxa used by Random Forest to distinguish FIV + and FIV - shelter cats from healthy house cats

Figure S1. Distributions of the relative abundances of thirty core bacterial genera in healthy pet cats. These were bacterial genera that were found in at least 55% of samples at a threshold of at least 25 reads per sample.

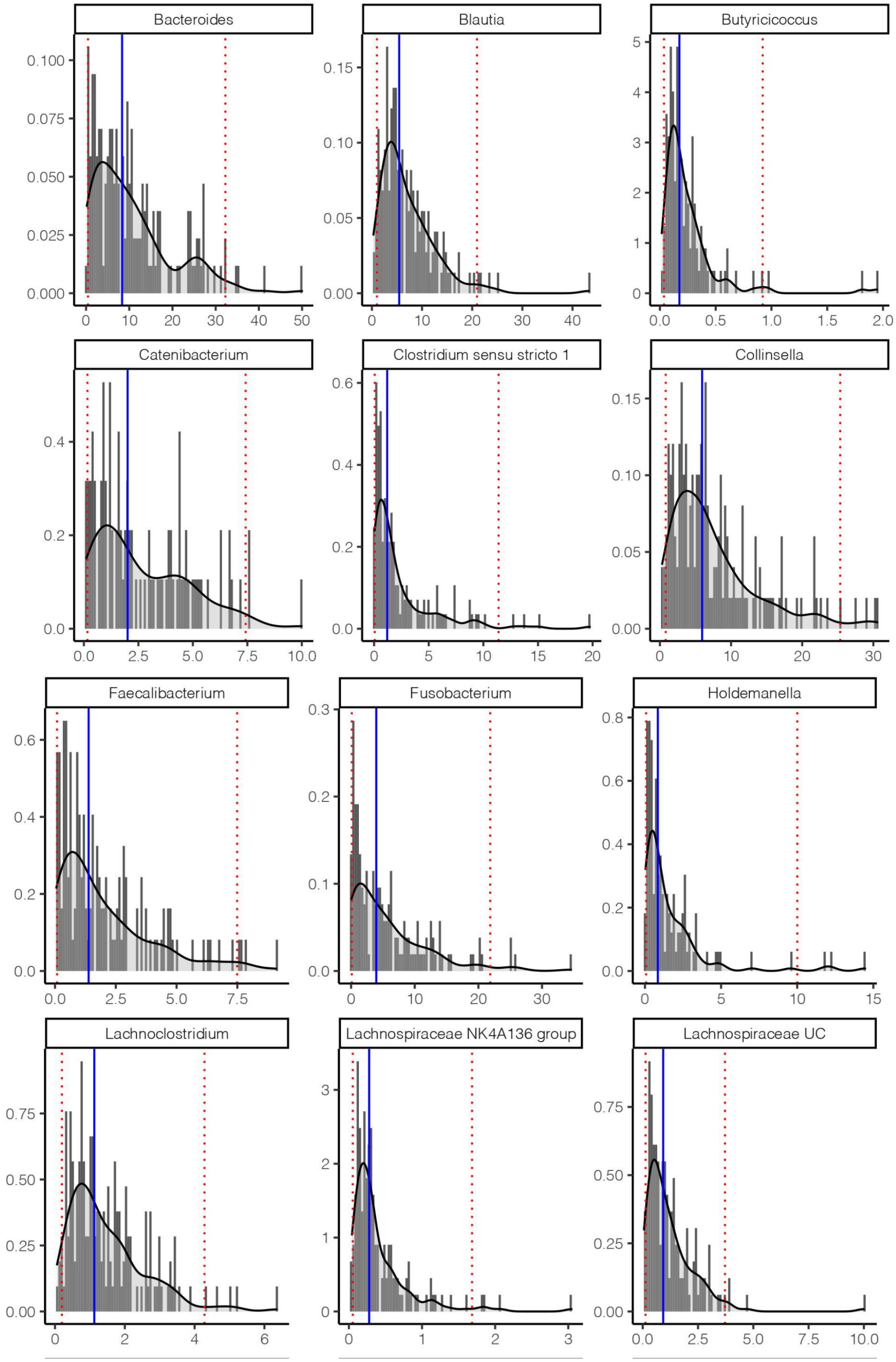

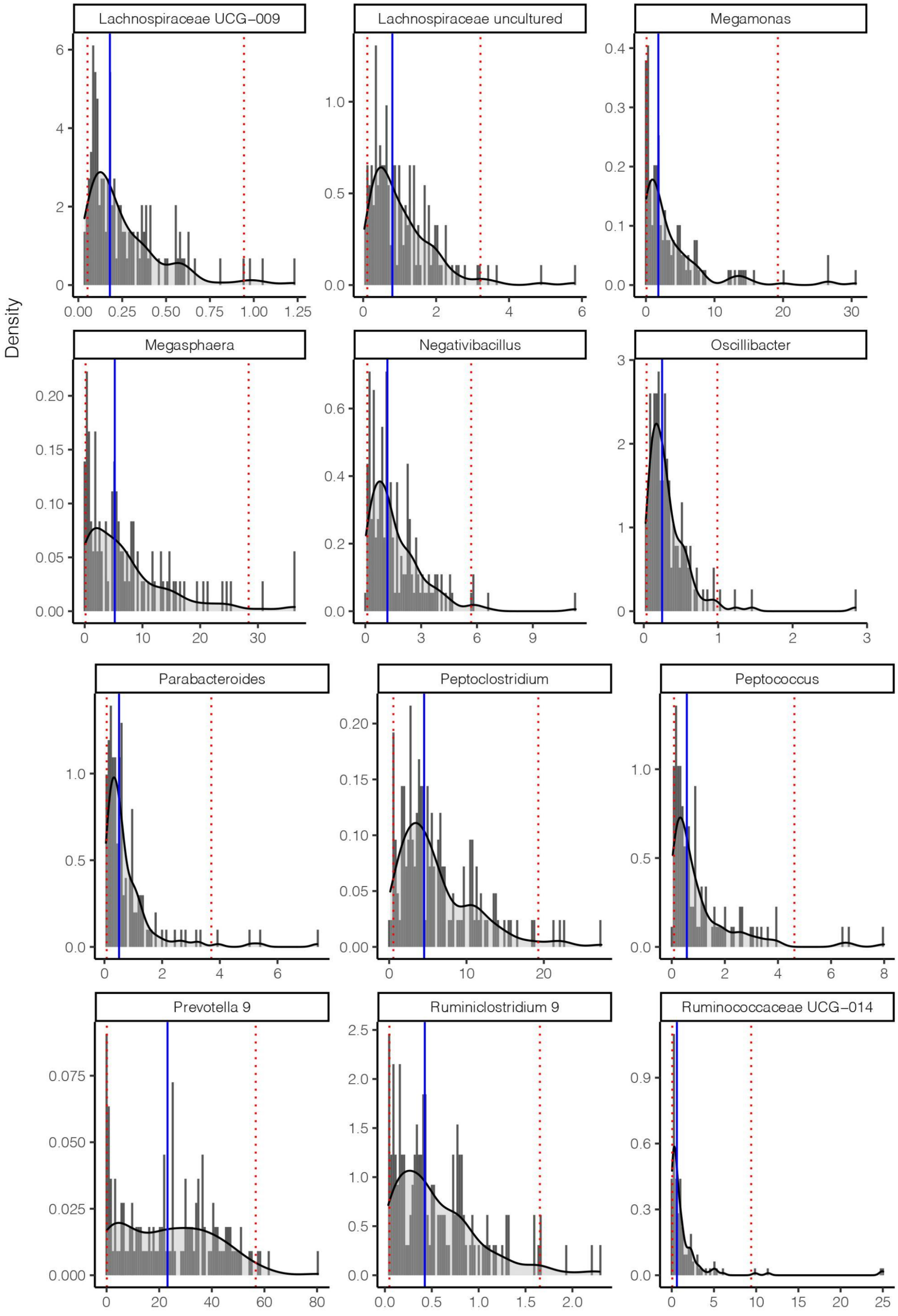

Lachnospiraceae UCG−009
Lachnospiraceae uncultured
Megamonas
Megasphaera
Negativibacillus
Oscillibacter
Parabacteroides
Peptoclostridium
Peptococcus
Prevotella 9
Ruminiclostridium 9
Ruminococcaceae UCG−014
Density

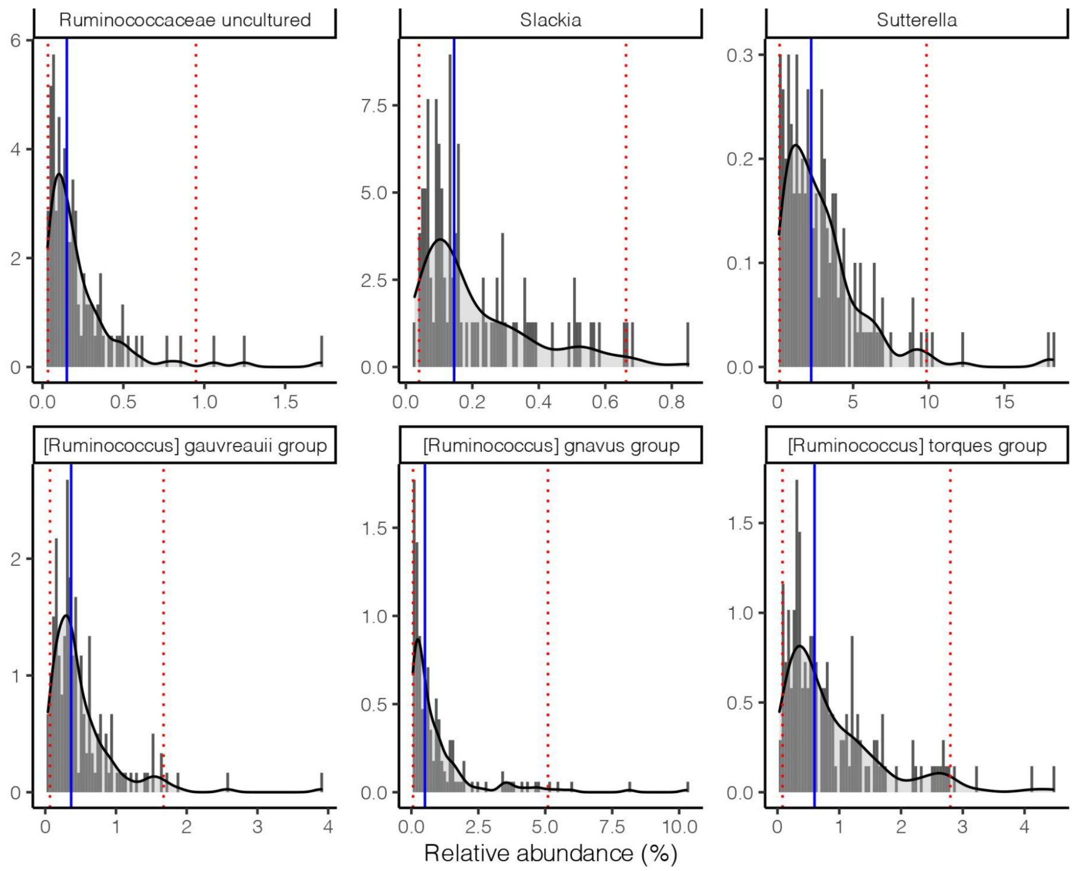

Ruminococcaceae uncultured
Slackia
Sutterella
[Ruminococcus] gauvreauii group
[Ruminococcus] gnavus group
[Ruminococcus] torques group
Relative abundance (%)

Figure S2. Three bacteria genera were found at lower relative abundances in older healthy cats compared to younger cats. We used MaAsLin2 for the analyses, set alpha to 0.05, and adjusted p.values for multiple comparisons using FDR.

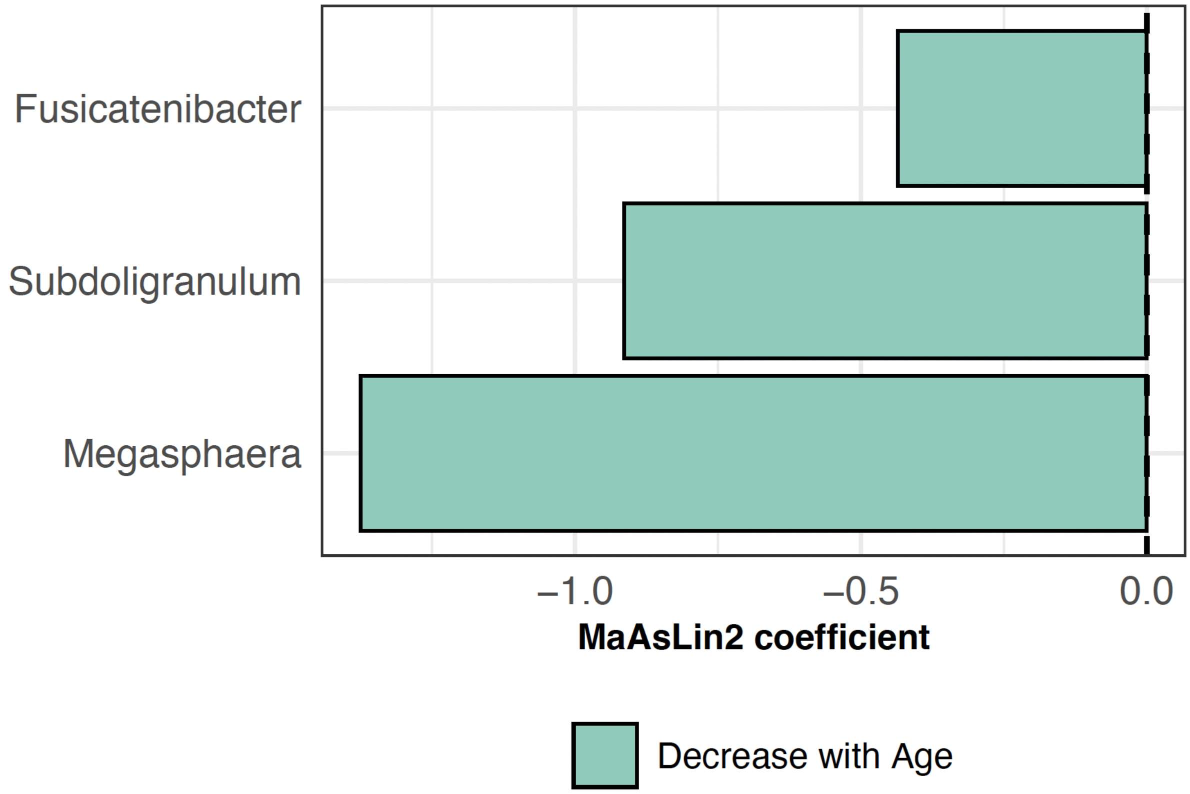

Figure S3. Bacterial genera enriched in the fecal microbiomes of cats that include dry food, raw food, or wet food in their diets. We used MaAsLin2 for the analyses, set alpha to 0.05, and adjusted p.values for multiple comparisons using FDR.

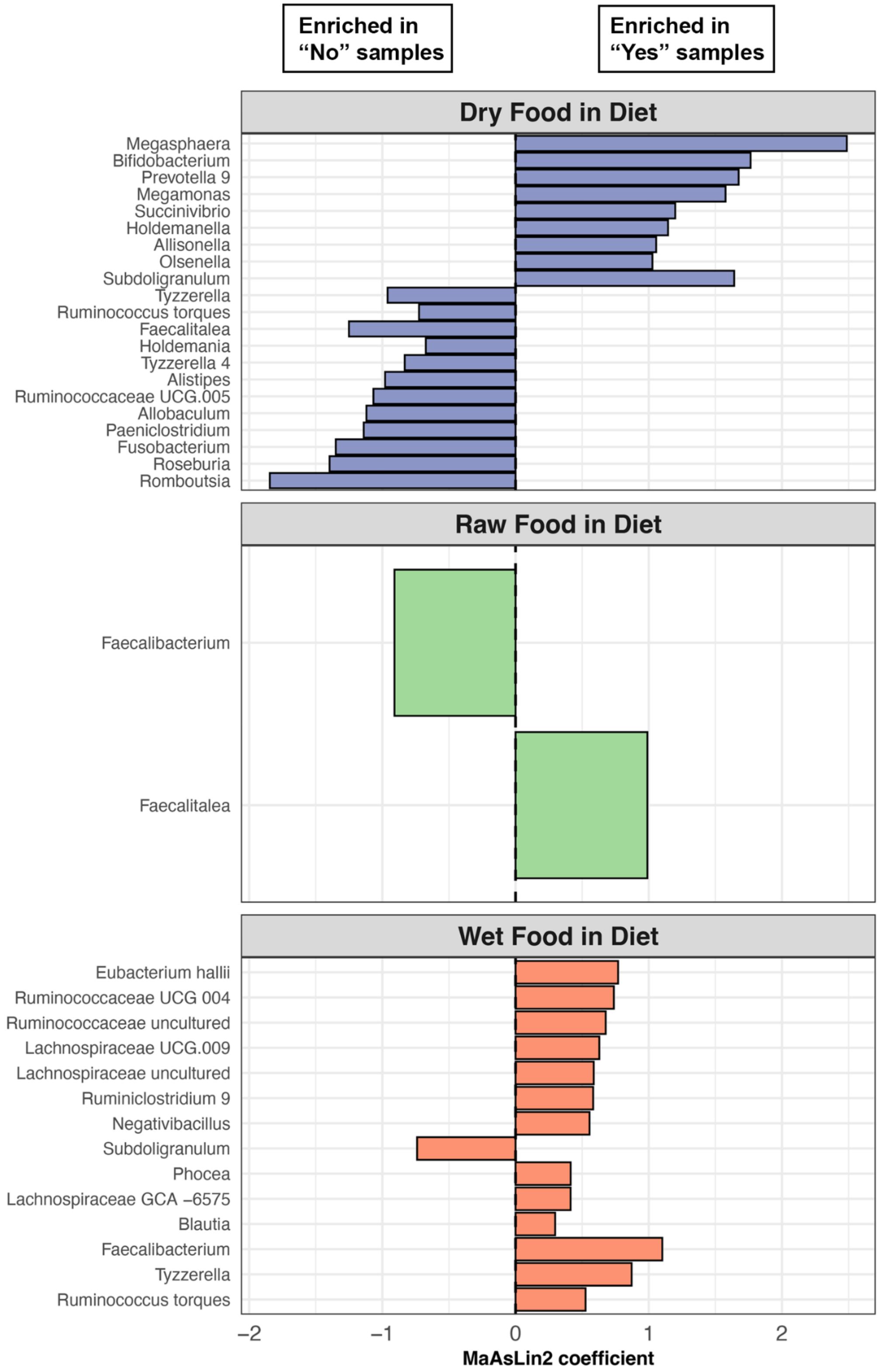

Figure S4. Random forest classifier accurately distinguished the fecal microbiomes of cats that include dry food in their diet from those that do not. Heatmap showing the logged frequencies of 24 bacterial genera by diet component. The 24 bacterial genera comprised half of the model's importance.

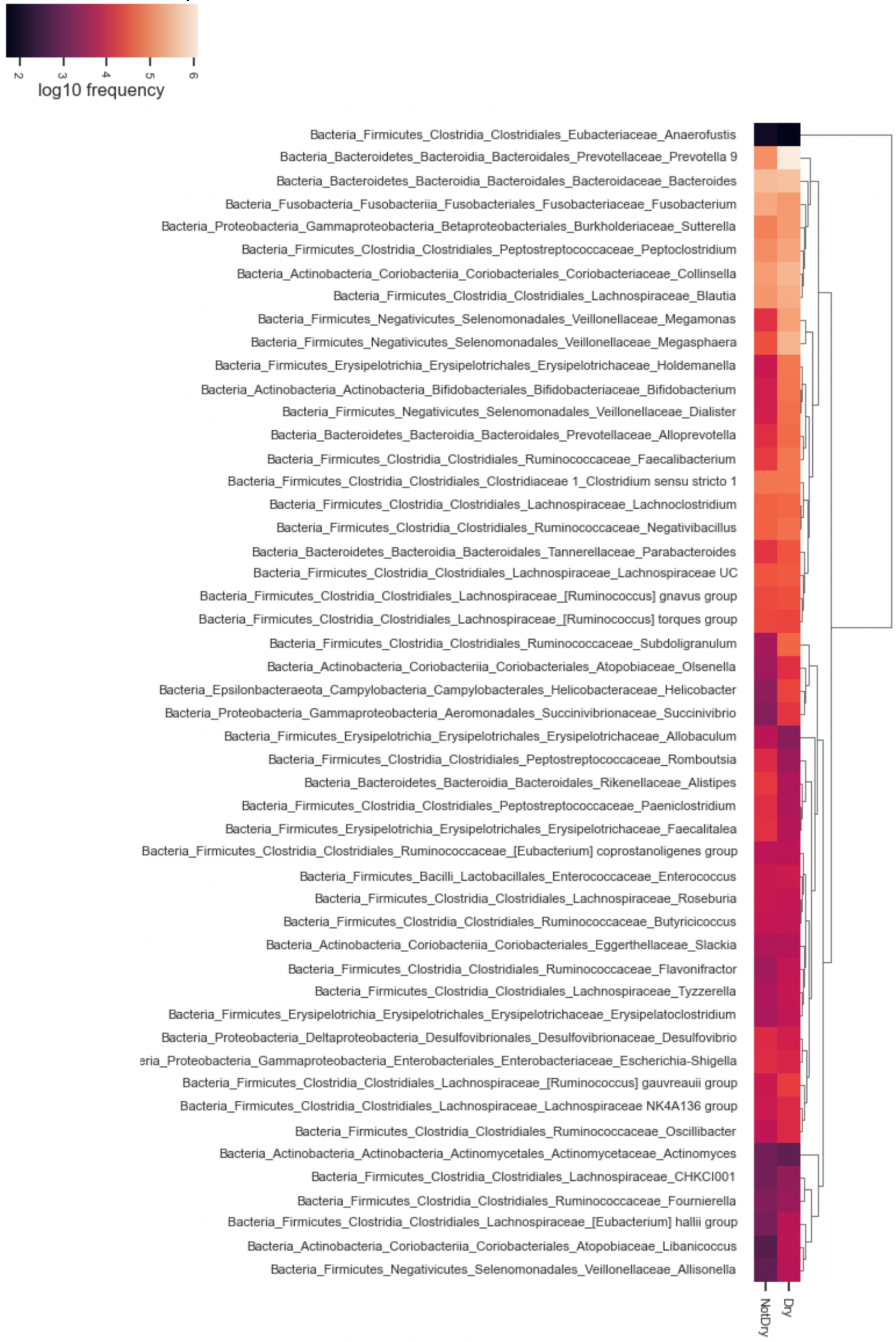

Figure S5. Random forest classifier accurately distinguished the fecal microbiomes of healthy house cats from those of FIV negative and FIV positive shelter cats. Heatmap showing the logged frequencies of 27 bacterial genera by living environment and FIV status. The 27 bacterial genera comprised half of the model's importance.

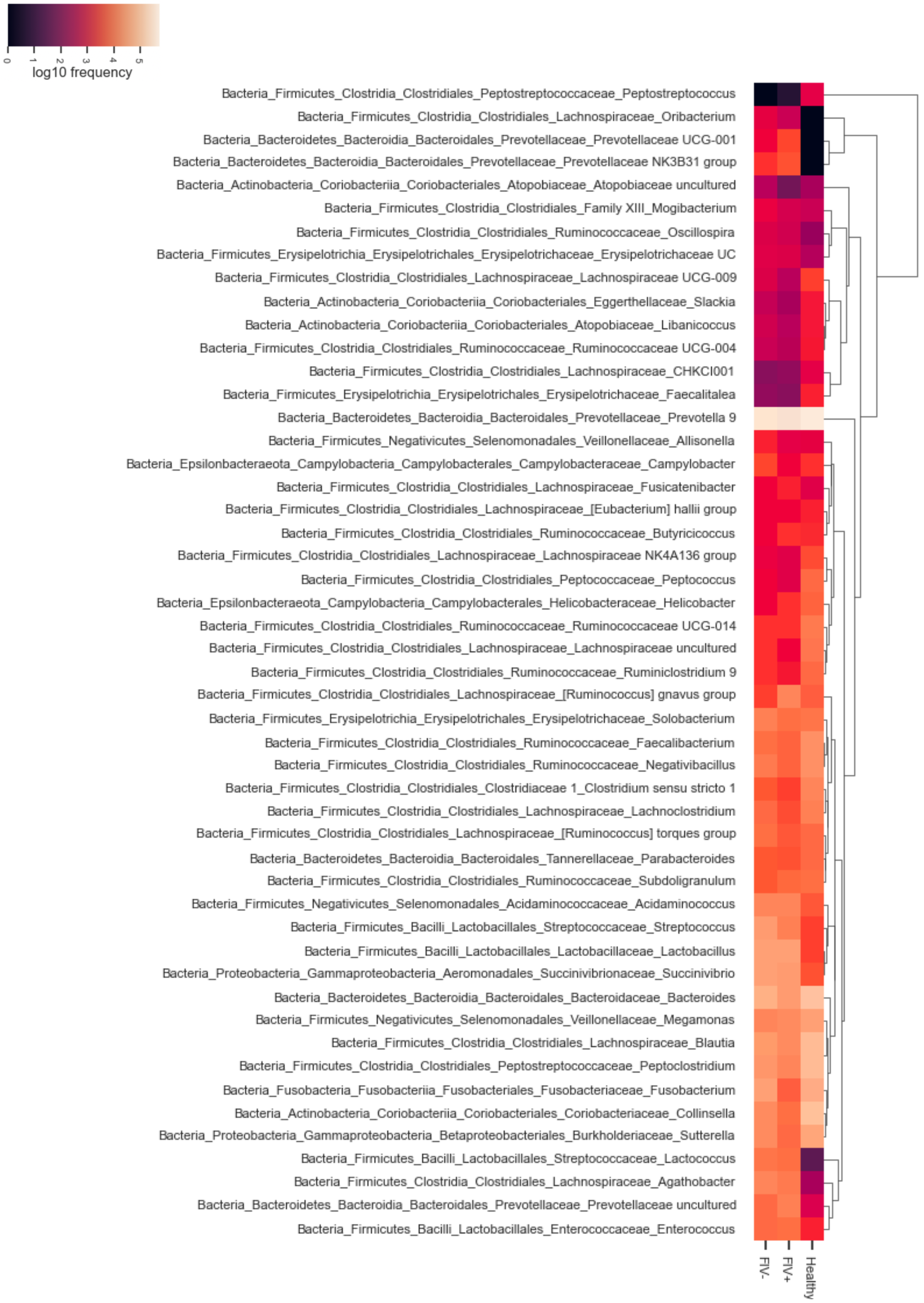

Supplement: Supplementary file 1 [file vetsci-09-00635-s001.zip › vetsci-1975773-Supplementary Figures.pdf]
